# Risk factors for community-acquired pneumonia in older adults in Japan after the introduction of the childhood PCV13

**DOI:** 10.1186/s12879-026-13820-7

**Published:** 2026-06-16

**Authors:** Naoki Inoshima, Kei Nakashima, Kanzo Suzuki, Hirohiko Nagasaka, Toshiaki Niwa, Chikara Nakahama, Naoyuki Miyashita, Satomi Iwamoto, Sakae Kan, Kyoko Kondo, Satoko Ohfuji, Wakaba Fukushima

**Affiliations:** 1 Department of Pulmonology, Kameda Medical Center, 929 Higashi-cho, Kamogawa, 296-8602 Chiba Japan; 2 Department of Community-based Medical Education, Nagoya City University Graduate School of Medical Sciences, Nagoya City University, Nagoya, Aichi Japan; 3 Nagasaka Clinic, Okazaki, Aichi Japan; 4 Hamada Asai Clinic, Tajimi, Gifu Japan; 5 Nakahama Clinic, Osaka, Osaka Japan; 6 First Department of Internal Medicine, Division of Respiratory Medicine, Infectious Disease and Allergology, Kansai Medical University, Hirakata, Osaka Japan; 7 Asuke Hospital, Toyota, Aichi Japan; 8 Kaisei Hospital, Nagoya, Aichi Japan; 9 OMU Core Facilities Life Sciences Section, Osaka Metropolitan University, Osaka, Osaka Japan; 10 Department of Public Health, Graduate School of Medicine, Osaka Metropolitan University, Osaka, Osaka Japan; 11 Research Center for Infectious Disease Sciences, Gruate School of Medicine, Osaka Metropolitan University, Osaka, Osaka Japan; 12 Osaka International Research Center for Infectious Diseases, Osaka Metropolitan University, Osaka, Osaka Japan

**Keywords:** Community-acquired pneumonia, Older adults, Risk factors

## Abstract

**Background:**

Community-acquired pneumonia (CAP) remains a leading cause of death globally. Although childhood 13-valent pneumococcal conjugate vaccine (PCV13) has altered the epidemiology of pneumococcal disease in children, risk factors for CAP in older adults remain insufficiently explored. Additionally, few studies have compared risks between those aged 65–74 and ≥ 75 years. We evaluated risk factors for CAP in older Japanese adults by age.

**Methods:**

We conducted a secondary analysis of data from a multicenter, nationwide case-control study conducted between October 2016 and December 2019. Cases were individuals aged ≥ 65 years with CAP. Up to five controls per case were matched by sex, fiscal-year age, and visit date. Clinical and lifestyle data were questionnaire-based. Adjusted odds ratios (aORs) were calculated for participants overall and stratified by age (65–74 and ≥ 75 years) using conditional logistic regression.

**Results:**

Analysis included 142 cases and 596 controls. CAP risk was associated with living with children ≤ 6 years (aOR: 6.15, 95% confidence interval [CI]: 2.84–13.32), low body mass index (BMI) (< 18.5 kg/m²) (aOR: 1.78, 95% CI: 1.02–3.09), and impaired activities of daily living (ADL) (aOR: 2.44, 95% CI: 1.12–5.30). High BMI (≥ 25.0 kg/m²) was associated with a reduced risk (aOR: 0.55, 95% CI: 0.31–0.95). Among those aged 65–74 years, living with children ≤ 6 years (aOR: 4.89, 95% CI: 1.83–13.08) was a risk factor for CAP, whereas high BMI (aOR: 0.41, 95% CI: 0.18–0.89) and gastrointestinal disease (aOR: 0.19, 95% CI: 0.04–0.84) were associated with a reduced risk. Among those aged ≥ 75 years, chronic obstructive pulmonary disease (aOR: 3.59, 95% CI: 1.29–9.93), asthma (aOR: 2.99, 95% CI: 1.25–7.16), living with children ≤ 6 years (aOR: 11.62, 95% CI: 3.12–43.33), and impaired ADL (aOR: 2.90, 95% CI: 1.10–7.65) were risk factors for CAP. Only living with children ≤ 6 years was significant for pneumococcal CAP (aOR: 7.22, 95% CI: 1.23–42.34).

**Conclusions:**

Living with children ≤ 6 years remains a major CAP risk factor in older adults after childhood PCV13 introduction. However, risk factors differ by age. Respiratory comorbidities and functional decline are dominant drivers of CAP in adults aged ≥ 75 years. Prevention strategies should consider age-specific dominant risk factors.

**Supplementary Information:**

The online version contains supplementary material available at 10.1186/s12879-026-13820-7.

## Background

Community-acquired pneumonia (CAP) is one of the major causes of hospitalization and mortality in older adults [1]. According to the World Health Organization, lower respiratory infections, including CAP, ranked as the fifth leading cause of death and, aside from COVID-19, remained the leading cause of communicable disease death worldwide in 2024 [2]. The high CAP morbidity and mortality rates in older adults have not improved for decades despite advances in medical care such as standardized guidelines, antimicrobial therapy, and prevention measures for high-risk populations [3–5]. Evaluating the risk factors for CAP is essential not only for implementing appropriate prevention and management strategies, but also for identifying areas for future pneumonia-related research and for determining key confounders in epidemiological studies, including vaccine studies [6, 7]. Previous systematic reviews have identified key risk factors for CAP including older age, male sex, smoking, environmental exposures, malnutrition, prior CAP, chronic bronchitis/chronic obstructive pulmonary disease (COPD), asthma, functional impairment, poor oral hygiene, immunosuppressive therapy, and use of gastric acid-suppressing drugs [8, 9].

Vaccination is critical for preventing pneumonia, with pneumococcal vaccination being the main prevention measure against *Streptococcus pneumoniae* infection [10]. The 23-valent pneumococcal polysaccharide vaccine (PPSV23) was the first pneumococcal vaccine used globally for older adults, and subsequently the 7-valent and 13-valent pneumococcal conjugate vaccines (PCV7 and PCV13) were introduced for children, with PCV13 later being introduced for adults [11]. Following the introduction of PCV13 in Japan in November 2013, vaccination coverage in children reached approximately 90% in 2014 [7]. PCV13 vaccination in children has transformed the epidemiology of pneumococcal disease, mainly by reducing vaccine-type pneumococcal carriage [12] and altering pneumococcal serotype distribution through serotype replacement [11, 13–15], although overall pneumococcal carriage may remain relatively unchanged [16]. Nevertheless, these changes may influence pneumococcal transmission dynamics and the serotype distribution of adult pneumococcal pneumonia. However, few studies have examined risk factors for CAP in older adults since the introduction of routine childhood PCV13 vaccination. In Spain PCV13 was approved for children in 2010 but vaccination coverage remained at approximately 50% until 2013. A study conducted in Spain between 2009 and 2010 identified HIV infection, COPD, asthma, smoking, and poor oral hygiene as significant risk factors for CAP in adults [17]. In Japan, after the introduction of PCV13 vaccination in children in 2013, a claims study using data collected from 2018 to 2022 identified impaired social functioning as a risk factor for adult CAP, although the use of administrative data limited the precision of the estimate [18]. Additionally, the risk of developing pneumonia has been reported to be higher in adults aged 75 years and older [19–22]. This suggests that, even within the older adult population aged ≥ 65 years, the risk may differ between those aged < 75 years and those aged ≥ 75 years. However, to our knowledge, no study has compared the risks in older adults aged < 75 years with those of older adults aged ≥ 75 years to evaluate risk factors for pneumonia onset.

Our group conducted a nationwide case-control study between 2016 and 2019 to evaluate the effectiveness of PPSV23 against CAP among older adults in Japan [7], at a time when PPSV23 coverage in older adults was approximately 55% [23] and PCV13 coverage in children was already high. In this case-control study, PPSV23 was not associated with a statistically significant protective effect against CAP, possibly due to serotype replacement owing to childhood PCV13 use and resulting changes in the serotype distribution [7]. Using data from this 2016–2019 study, we performed a secondary analysis to identify risk factors for CAP in older Japanese adults (aged ≥ 65 years) after the introduction of childhood PCV13 and conducted subgroup analyses stratified by age (< 75 years and ≥ 75 years).

## Methods

### Study design

We conducted a secondary analysis of data from a nationwide, multicenter, prospective, case-control study conducted in Japan. The original research was a hospital-based matched case-control study carried out from October 1, 2016, to December 31, 2019, at 41 healthcare facilities, which included 30 hospitals and 11 clinics, spread across the Hokkaido, Tohoku, Hokuriku, Kanto, Tokai, Kinki, Shikoku, and Kyushu regions [7]. Cases included in this study were restricted to adults aged 65 years and older who were living at home and developed pneumonia, and the outcomes focused on CAP. Because the cases were patients diagnosed with CAP in outpatient settings, including emergency departments in clinics or hospitals, controls were chosen from outpatients attending the same clinics or hospitals. In October 2014, a national immunization program for PPSV23 targeting individuals aged 65 years and older was launched, with vaccinations provided to eligible participants at local clinics or hospitals. In this program, PPSV23 was administered to adults who reached 65, 70, 75, 80, 85, 90, 95, or 100 years of age within a given fiscal year. As transitional measures, individuals aged ≥ 100 years at the end of fiscal year 2013 were additionally eligible in fiscal year 2014 at the time of program introduction. Similarly, those aged ≥ 100 years at the end of fiscal year 2018 were eligible in fiscal year 2019 when the transitional measures were extended. Ethical approval for this secondary analysis was obtained from the central institutional review board at Osaka Metropolitan University (#2023-082). In addition, the study was approved by Kameda Medical Center and Nagoya City University, which were participating institutions. The research adhered to the principles outlined in the Declaration of Helsinki. The institutional review board waived the requirement for informed consent, and patients were given the opportunity to opt out of the study.

### Definition of cases and controls

In the original study [7], conducted between October 1, 2016, and September 30, 2019, individuals aged 65 years and older who were newly diagnosed with CAP at outpatient facilities, including emergency departments, were prospectively enrolled as cases. These patients were registered on the day that they were diagnosed with pneumonia in either the outpatient or emergency department. The physicians at the participating institutions diagnosed pneumonia based on clinical manifestations such as fever, cough, and sputum, along with elevated leukocyte counts or C-reactive protein levels, and the presence of infiltration on chest X-ray or computed tomography. The diagnosis of pneumococcal pneumonia was subsequently confirmed through sputum culture using a semiquantitative method, Gram staining showing Gram-positive cocci in pairs, blood culture, or a positive pneumococcal urinary antigen test. For each case, we recruited up to five controls without pneumonia who visited the same clinics and hospitals, matched by sex, fiscal-year age (corresponding to the age categories of the national immunization program), and visit date (within 3 months of confirmation of the corresponding case). The exclusion criteria for both cases and controls included residing in a nursing home, having aspiration pneumonia (caused by inhalation during eating or vomiting), having malignant tumors, taking oral steroids or immunosuppressants, or having a history of splenectomy. Patients with malignancies were excluded owing to potential immunosuppression. To minimize overrepresentation in the number of cases at some facilities, each facility was limited to enrolling a maximum of five matched sets (five cases and 25 controls) per year.

### Data collection

For current analysis, we extracted variables collected in the original study [7]. In the original study, the attending physician gathered clinical details on both cases and controls using a structured questionnaire, which included information on: (a) self-reported age, sex, date of birth, presence or absence of underlying respiratory diseases (such as chronic bronchitis, pulmonary emphysema, interstitial pneumonia, asthma, pulmonary tuberculosis sequelae, or other respiratory diseases), and vaccination history (dates of PPSV23, PCV13, and influenza vaccine administration); (b) pneumonia-specific information (for cases only), such as the date of diagnosis, clinical manifestations, laboratory data related to the pneumonia diagnosis (leukocyte count and C-reactive protein levels), and results from diagnostic tests (pneumococcal urinary antigen test and sputum culture). Additionally, we extracted data from a self-administered questionnaire that study participants or their next of kin were asked to fill out. This questionnaire included details regarding their status at the time of enrollment, such as height, body weight, living with children aged under 6 years old, activities of daily living (ADL; bedridden, semi-bedridden, semi-self-supported, or self-supported), presence or absence of underlying respiratory diseases (such as chronic bronchitis, pulmonary emphysema, interstitial pneumonia, asthma, pulmonary tuberculosis sequelae, or other respiratory diseases), presence or absence of underlying diseases (hypertension, dyslipidemia, chronic kidney disease, heart disease, stroke, diabetes mellitus, liver disease, or gastrointestinal disease), alcohol use history (never, past, occasionally, or daily), smoking history (never smoker, past smoker, or current smoker) and vaccination status (PPSV23 in the past 5 years and influenza vaccination in the past 6 months). Both the physician-administered questionnaire and the self-administered questionnaire were developed specifically for the original study [7] and have not been published elsewhere. An English-language version of the questionnaires is provided in Supplementary Material 1. Respiratory disease status was collected in both the questionnaires. The primary analysis used physician-administered respiratory disease status, which was followed by a sensitivity analysis that uses the self-administered respiratory disease status. Pneumococcal vaccination status (i.e., vaccinated or unvaccinated) and the specific vaccine type were determined using information from two sources: data gathered by the attending physician and data reported by the participant or their next of kin. A participant was classified as “vaccinated” if either source indicated a history of vaccination, irrespective of the date of administration. For those classified as vaccinated, the specific vaccine type administered (e.g., PPSV23, PCV13) was determined primarily based on the physician’s records. Influenza vaccination status was determined primarily from the information given in participant’s self-administered questionnaire. When vaccination status within the past 6 months was unclear, physician’s records were used for verification. Participants were classified as vaccinated if either source indicated influenza vaccination within 6 months prior to the case’s diagnosis date or the control’s enrollment date.

### Statistical analysis

In this secondary analysis, participants with any missing data were excluded using listwise deletion. All underlying diseases were categorized as either present or absent, whereas ADL status was classified as either “not independent” (bedridden, semi-bedridden, or semi-self-supported) or “independent” (self-supported) for the purpose of analysis. Body mass index (BMI) was divided into three categories: <18.5, 18.5–24.9, and ≥ 25.0 kg/m^2^. The Wilcoxon rank-sum test, Chi-square test, and Fisher’s exact test were used to assess the statistical significance of differences between the case and control groups, as appropriate. Conditional logistic regression models were used to calculate odds ratios (ORs) and 95% confidence intervals (CIs) for each factor associated with pneumonia, using physician-administered respiratory disease status as the main exposure variable for underlying respiratory conditions. Both crude and adjusted ORs were estimated. Adjustment variables included established risk factors for pneumonia (smoking, COPD, asthma, diabetes mellitus, ADL) [8, 9], variables with *p* < 0.20 in the comparison of baseline characteristics, and history of pneumococcal and influenza vaccination. Subgroup analyses stratified by fiscal-year age (< 75 years and ≥ 75 years) were also conducted using conditional logistic regression. While fiscal-year age was used for stratification to maintain the validity of the conditional logistic model in accordance with the matching criteria, chronological age was reported in the results for intuitive interpretation. The same adjustment variables as in the overall analysis were used in all subgroup models. We repeated the primary and age-stratified conditional logistic regression analyses as a sensitivity analysis, using self-administered respiratory disease status instead of physician-administered respiratory disease status. This analysis was performed to assess the robustness of the main findings, given the possibility that physician-administered respiratory disease status could be influenced by diagnostic evaluations performed at the time of CAP diagnosis. Participants with missing data in the self-administered respiratory disease variables were excluded from this sensitivity analysis.

Finally, we performed an additional conditional logistic regression analysis limited to cases with pneumococcal CAP because routine childhood PCV13 vaccination can reduce vaccine-type pneumococcal carriage and alter serotype distribution, potentially modifying the association between living with children aged ≤ 6 years and pneumococcal CAP in older adults. Although the same adjustment variables were used as in the primary model, variables with zero events in at least one group (i.e., gastrointestinal disease and liver disease) were excluded to avoid quasi-complete separation and to ensure model stability. Age-stratified multivariable analyses were not performed for pneumococcal CAP because the limited number of cases (*n* = 31) would have resulted in multiple covariates with zero events within age strata, causing quasi-complete separation and non-estimable odds ratios. We also repeated the pneumococcal CAP analysis as a sensitivity analysis using self-administered respiratory disease status instead of physician-administered respiratory disease status. All statistical analyses were performed using SAS version 9.4 (SAS Institute, Inc., Cary, NC, USA), with statistical significance set at *p* < 0.05.

## Results

**Fig. 1:**
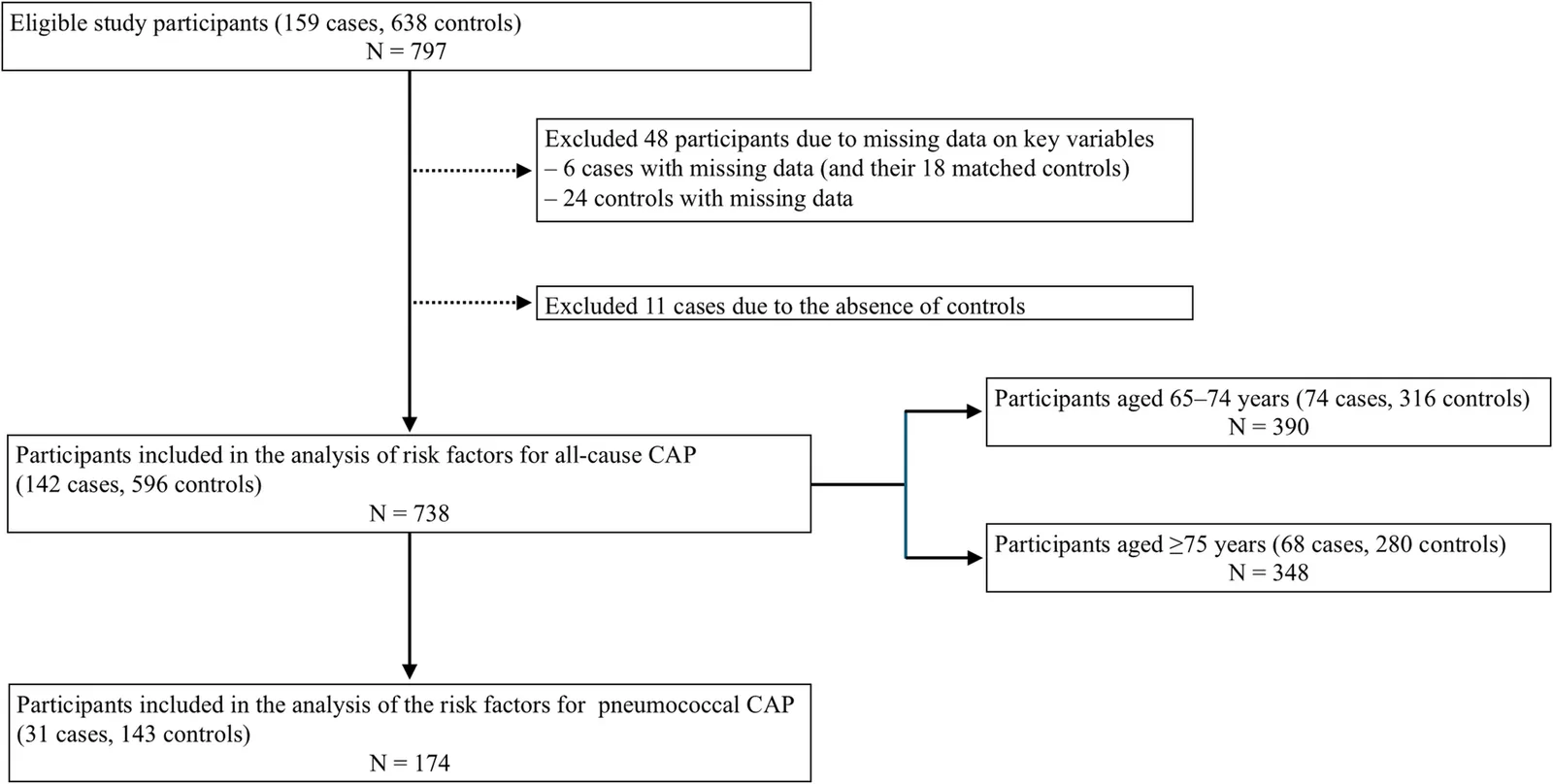
Flowchart showing participant selection

Figure 1 shows the flow of study participants. Of the 797 eligible participants (159 cases and 638 controls), 59 were excluded from the analysis due to missing data or a lack of matched controls, leaving 738 participants (142 cases and 596 controls) included in the analysis of risk factors for all-cause CAP. Among the cases, 102 were recruited from hospital-based internal medicine departments, 39 from clinics, and 1 from an unspecified department within a hospital. Among the controls, 342 were enrolled from hospital-based internal medicine departments, 185 from clinics, and 69 from non-internal medicine or unspecified departments of hospitals, including orthopedics (*n* = 25), ophthalmology (*n* = 10), otorhinolaryngology (*n* = 6), dermatology (*n* = 4), urology (*n* = 3), surgery (*n* = 2), gynecology (*n* = 1), and unspecified (*n* = 18) departments. This analysis population was stratified into two subgroups based on fiscal-year age: the subgroup aged 65–74 years (*N* = 390) included 74 cases and 316 controls, and the subgroup aged ≥ 75 years (*N* = 348) included 68 cases and 280 controls. Additionally, 174 participants, comprising 31 cases and 143 controls were included in the analysis of risk factors for pneumococcal CAP.

Table 1 shows the baseline characteristics of all participants. The median age was 75 years in both the case and control groups. The proportion of male participants was 56% in the case group and 53% in the control group. The case and control groups had similar influenza vaccination (42% vs. 44%, respectively; *p* = 0.603) and pneumococcal vaccination (58% vs. 56%, respectively; *p* = 0.603) coverage. Among underlying medical conditions, COPD was significantly more prevalent in the case group (24% vs. 14%; *p* = 0.003), whereas dyslipidemia was more prevalent in the control group (19% vs. 29%; *p* = 0.014). Additionally, the proportion of participants with a low BMI (< 18.5 kg/m²) was significantly higher in the case group than in the control group (23% vs. 11%; *p* < 0.001). Cases were also more likely to living with children aged ≤ 6 years (15% vs. 3%; *p* < 0.001) and to have impaired ADL (defined as “not independent”) (10% vs. 5%; *p* = 0.014). Alcohol consumption and the prevalence of diabetes mellitus did not differ significantly between the case and the control groups.

**Table 1:** Baseline characteristics of participants (*N* = 738)

Characteristics	Cases (*N* = 142)	Controls (*N* = 596)	*p* ^b^
Age (years),^a^ median (IQR)	75 (70–80)	75 (70–80)	0.981
Sex			
Male	79 (56%)	315 (53%)	0.550
Female	63 (44%)	281 (47%)	
Vaccination			
Pneumococcal vaccine	83 (58%)	334 (56%)	0.603
PPSV23 only	79 (56%)	310 (52%)	
PCV13 only	1 (0.7%)	10 (1.7%)	
Both	1 (0.7%)	11 (1.9%)	
Unknown	2 (1.4%)	3 (0.5%)	
Influenza vaccine	59 (42%)	262 (44%)	0.603
Respiratory diseases			
COPD	34 (24%)	83 (14%)	0.003
Interstitial pneumonia	6 (4%)	24 (4%)	0.914
Bronchial asthma	23 (16%)	74 (12%)	0.231
Tuberculosis (sequelae)	5 (4%)	9 (2%)	0.161
Underlying diseases			
Hypertension	67 (47%)	320 (54%)	0.163
Dyslipidemia	27 (19%)	174 (29%)	0.014
Diabetes	35 (25%)	138 (23%)	0.706
Chronic kidney disease	9 (6%)	54 (9%)	0.297
Heart disease	19 (13%)	103 (17%)	0.261
Stroke	6 (4%)	27 (5%)	0.875
Gastrointestinal disease	13 (9%)	84 (14%)	0.118
Liver disease	3 (2%)	28 (5%)	0.168
BMI (kg/m²)			
< 18.5	33 (23%)	64 (11%)	< 0.001
18.5–24.9	87 (61%)	372 (62%)	
≥ 25.0	22 (15%)	160 (27%)	
Living with children aged ≤ 6 years			
No	121 (85%)	580 (97%)	< 0.001
Yes	21 (15%)	16 (3%)	
Activities of daily living			
Independent	128 (90%)	569 (95%)	0.013
Not independent	14 (10%)	27 (5%)	
Alcohol use history			
Never/Past	88 (62%)	374 (63%)	0.852
Occasionally	27 (19%)	102 (17%)	
Daily	27 (19%)	120 (20%)	
Smoking history			
Never smoker	75 (53%)	343 (58%)	0.203
Past smoker	49 (35%)	205 (34%)	
Current smoker	18 (13%)	48 (8%)	

^a^ Age indicates chronological age at the time of diagnosis or enrollment

^b^ The p values were calculated using the Wilcoxon rank-sum test, chi-square test, or Fisher’s exact test, as appropriate

BMI, body mass index; COPD, chronic obstructive pulmonary disease; IQR, interquartile range

Table 2 shows the ORs for CAP. In the unstratified adjusted analysis, low BMI (< 18.5 kg/m²; adjusted OR [aOR] 1.78, 95% CI, 1.02–3.09), living with children aged ≤ 6 years (aOR 6.15, 95% CI, 2.84–13.32), and impaired ADL (aOR 2.44, 95% CI, 1.12–5.30) were significantly associated with an increased risk of pneumonia, whereas a high BMI (≥ 25.0 kg/m²) was associated with a decreased risk (aOR 0.55, 95% CI, 0.31–0.95). Pneumococcal and influenza vaccination were not significantly associated with all-cause CAP in the adjusted models.

**Table 2:** Crude and adjusted ORs for CAP in older adults, overall and stratified by age group

	Overall (65 ≥ years) (*N* = 738)		65–74 years (*N* = 390)		≥ 75 years (*N* = 348)	
	Crude OR (95% CI)	Adjusted OR* (95% CI)	Crude OR (95% CI)	Adjusted OR* (95% CI)	Crude OR (95% CI)	Adjusted OR* (95% CI)
COPD						
No	1	1	1	1	1	1
Yes	1.91 (1.13–3.20)	1.69 (0.92–3.08)	1.60 (0.82–3.13)	1.33 (0.61–2.94)	2.51 (1.09–5.77)	3.59 (1.29–9.93)
Bronchial asthma						
No	1	1	1	1	1	1
Yes	1.45 (0.86–2.44)	1.37 (0.78–2.41)	0.99 (0.47–2.08)	0.71 (0.31–1.64)	2.22 (1.05–4.66)	2.99 (1.25–7.16)
Tuberculosis (sequelae)						
No	1	1	1	1	1	1
Yes	2.68 (0.86–8.31)	2.40 (0.71–8.15)	1.67 (0.17–16.02)	2.11 (0.18–25.38)	3.23 (0.85–12.30)	2.21 (0.50–9.76)
Hypertension						
No	1	1	1	1	1	1
Yes	0.76 (0.51–1.13)	0.85 (0.55–1.33)	0.75 (0.44–1.28)	0.86 (0.47–1.60)	0.77 (0.43–1.39)	0.94 (0.47–1.89)
Dyslipidemia						
No	1	1	1	1	1	1
Yes	0.59 (0.35–0.97)	0.78 (0.45–1.35)	0.52 (0.26–1.06)	0.66 (0.31–1.41)	0.67 (0.32–1.39)	0.83 (0.36–1.94)
Diabetes						
No	1	1	1	1	1	1
Yes	1.04 (0.67–1.60)	1.36 (0.84–2.19)	0.80 (0.43–1.48)	1.05 (0.52–2.11)	1.36 (0.74–2.51)	1.82 (0.90–3.69)
Gastrointestinal disease						
No	1	1	1	1	1	1
Yes	0.58 (0.30–1.12)	0.59 (0.29–1.19)	0.27 (0.07–0.99)	0.19 (0.04–0.84)	0.84 (0.39–1.82)	1.11 (0.46–2.68)
Liver disease						
No	1	1	1	1	1	1
Yes	0.41 (0.12–1.43)	0.37 (0.10–1.34)	0.22 (0.03–1.72)	0.14 (0.02–1.28)	0.74 (0.15–3.66)	0.78 (0.15–4.09)
BMI (kg/m²)						
< 18.5	2.12 (1.29–3.46)	1.78 (1.02–3.09)	1.90 (0.93–3.86)	1.59 (0.69–3.62)	2.34 (1.18–4.63)	1.97 (0.86–4.50)
18.5–24.9	1	1	1	1	1	1
≥ 25.0	0.55 (0.32–0.93)	0.55 (0.31–0.95)	0.43 (0.21–0.89)	0.41 (0.18–0.89)	0.73 (0.34–1.58)	0.58 (0.25–1.36)
Living with children aged ≤ 6 years						
No	1	1	1	1	1	1
Yes	6.66 (3.23–13.74)	6.15 (2.84–13.32)	6.53 (2.54–16.78)	4.89 (1.83–13.08)	6.85 (2.22–21.14)	11.62 (3.12–43.33)
Activities of daily living						
Independent	1	1	1	1	1	1
Not independent	2.12 (1.04–4.33)	2.44 (1.12–5.30)	2.51 (0.68–9.23)	3.86 (0.78–19.20)	1.98 (0.85–4.62)	2.90 (1.10–7.65)
Smoking history						
Never smoker	1	1	1	1	1	1
Past smoker	1.15 (0.68–1.95)	1.07 (0.59–1.95)	1.32 (0.66–2.62)	1.22 (0.56–2.66)	0.98 (0.42–2.25)	0.73 (0.26–2.09)
Current smoker	1.84 (0.91–3.71)	1.58 (0.73–3.40)	1.43 (0.59–3.46)	1.11 (0.42–2.90)	2.98 (0.94–9.47)	2.40 (0.61–9.38)
Pneumococcal vaccine						
No	1	1	1	1	1	1
Yes	1.17 (0.79–1.76)	1.23 (0.82–2.04)	1.23 (0.70–2.18)	1.40 (0.72–2.70)	1.12 (0.63–1.97)	1.12 (0.57–2.20)
Influenza vaccine						
No	1	1	1	1	1	1
Yes	0.83 (0.53–1.30)	0.77(0.47–1.27)	0.98 (0.54–1.79)	0.86 (0.43–1.73)	0.68 (0.35–1.32)	0.67 (0.32–1.43)

*The model incorporated all variables listed in the Table

BMI, body mass index; CAP, community-acquired pneumonia; CI, confidence interval; COPD, chronic obstructive pulmonary disease; OR, odds ratio

Next, we conducted a subgroup analysis stratified by fiscal-year age (< 75 years vs. ≥75 years). In the subgroup of participants aged 65–74 years (Supplementary Table 1), dyslipidemia was less common among cases than among controls (18% vs. 29%; *p* = 0.039). Compared with controls, cases were more likely to have a BMI < 18.5 kg/m² (22% vs. 10%; *p* = 0.003) and more likely to be living with children aged ≤ 6 years (18% vs. 3%; *p* < 0.001). In the subgroup of adults aged 65–74 years, multivariable analysis (Table 2) showed that living with children aged ≤ 6 years was strongly associated with an increased risk of CAP (adjusted OR 4.89, 95% CI 1.83–13.08). In contrast, gastrointestinal disease (adjusted OR 0.19, 95% CI 0.04–0.84) and a higher BMI (≥ 25.0 kg/m²) (adjusted OR 0.41, 95% CI 0.18–0.89) were both associated with a lower risk of CAP.

In the subgroup of participants aged ≥ 75 years (Supplementary Table 2), cases were more likely than controls to have COPD (24% vs. 13%; *p* = 0.021), a BMI < 18.5 kg/m² (25% vs. 11%; *p* = 0.012), and to be living with children aged ≤ 6 years (12% vs. 2%; *p* < 0.001). In the subgroup aged ≥ 75 years, multivariable analysis (Table 2) showed that COPD (adjusted OR 3.59, 95% CI 1.29–9.93), bronchial asthma (adjusted OR 2.99, 95% CI 1.25–7.16), living with children aged ≤ 6 years (adjusted OR 11.62, 95% CI 3.12–43.33), and impaired ADL (adjusted OR 2.90, 95% CI 1.10–7.65) were associated with a significantly increased risk of CAP.

The sensitivity analysis using self-administered respiratory disease status included 686 participants (Supplementary Fig. 1). Baseline characteristics of the participants included in this sensitivity analysis are shown in Supplementary Table 3, and for those stratified by age group are shown in Supplementary Tables 4 and 5. The findings of the sensitivity analysis were generally consistent with those of the primary analysis. As shown in Supplementary Table 6, low BMI and living with children aged ≤ 6 years were associated with an increased risk of CAP in the overall population. In the subgroup aged 65–74 years, living with children aged ≤ 6 years remained associated with an increased risk of CAP, whereas gastrointestinal disease was associated with a lower risk. In the subgroup aged ≥ 75 years, COPD, bronchial asthma, living with children aged ≤ 6 years, and impaired ADL were associated with an increased risk of CAP. However, high BMI was no longer significantly associated with a reduced risk of CAP in the overall population or in the subgroup aged 65–74 years.

The baseline characteristics of cases (*n* = 31) with pneumococcal CAP and their matched controls (*n* = 143) are shown in supplementary Tables 7 and the adjusted ORs for this subgroup are shown in Table 3. Compared with controls, cases were more likely to be living with children aged ≤ 6 years (16% vs. 2%; *p* = 0.005). After adjustment, living with children aged ≤ 6 years remained a significant risk factor for pneumococcal CAP (adjusted OR 7.22, 95% CI 1.23–42.34). Conversely, neither pneumococcal nor influenza vaccination was significantly associated with pneumococcal CAP in the adjusted model. Baseline characteristics and ORs for the sensitivity analysis using self-administered respiratory disease status among participants included in the pneumococcal CAP analysis are shown in Supplementary Tables 8 and 9, respectively. Living with children aged ≤ 6 years remained associated with an increased risk of pneumococcal CAP.

**Table 3:** Crude and adjusted ORs for pneumococcal CAP (*N* = 174)

	Crude OR (95% CI)	Adjusted OR* (95% CI)
COPD		
No	1	1
Yes	1.09 (0.21–5.55)	0.65 (0.09–4.70)
Bronchial asthma		
No	1	1
Yes	2.05 (0.77–5.47)	2.21 (0.74–6.59)
Tuberculosis (sequelae)		
No	1	1
Yes	6.53 (0.56–76.72)	5.53 (0.37–83.53)
Hypertension		
No	1	1
Yes	0.68 (0.29–1.61)	0.73 (0.28–1.90)
Dyslipidemia		
No	1	1
Yes	0.38 (0.12–1.25)	0.43 (0.11–1.68)
Diabetes		
No	1	1
Yes	0.92 (0.39–2.17)	0.97 (0.33–2.84)
BMI (kg/m²)		
< 18.5	1.20 (0.38–3.76)	1.01 (0.25–4.10)
18.5–24.9	1	1
≥ 25.0	0.41 (0.13–1.25)	0.57 (0.18–1.82)
Living with children aged ≤ 6 years		
No	1	1
Yes	10.87 (2.08–56.71)	7.22 (1.23–42.34)
Activities of daily living		
Independent	1	1
Not independent	1.29 (0.26–6.50)	2.14 (0.35–13.03)
Smoking history		
Never smoker	1	1
Past smoker	1.20 (0.33–4.34)	1.17 (0.25–5.46)
Current smoker	2.97 (0.78–11.34)	2.00 (0.44–9.11)
Pneumococcal vaccine		
No	1	1
Yes	0.83 (0.37–1.89)	1.04 (0.36–3.03)
Influenza vaccine		
No	1	1
Yes	0.72 (0.26–2.06)	0.87 (0.23–3.28)

*The model incorporated all variables listed in the table

BMI, body mass index; CAP, community-acquired pneumonia; CI, confidence interval; COPD, chronic obstructive pulmonary disease; OR, odds ratio

## Discussion

In this secondary analysis of a multicenter case-control study conducted in Japan following the introduction of the PCV13 for children, we identified distinct risk factors for CAP among older adults. In all participants, living with children aged ≤ 6 years, low BMI, and impaired ADL were associated with an increased risk of CAP, whereas a high BMI was associated with a reduced risk. Among participants aged 65–74 years, living with children aged ≤ 6 years was the strongest risk factor, whereas high BMI and the presence of gastrointestinal disease were associated with a significantly reduced risk of CAP. In contrast, among those aged ≥ 75 years, underlying respiratory comorbidities, specifically COPD and bronchial asthma, and impaired ADL emerged as independent risk factors for CAP. Furthermore, living with children aged ≤ 6 years remained a potent risk factor in this older age group and was a significant risk factor for CAP. Additionally, in the analysis limited to pneumococcal CAP, only living with children aged ≤ 6 years was significantly associated with the outcome. These findings were largely supported by the sensitivity analysis using self-administered respiratory disease status. Taken together, our results suggest that although intergenerational transmission remains a critical pathway regardless of age, the contribution of respiratory comorbidities and functional decline becomes increasingly important as risk factors in individuals aged ≥ 75 years.

One of the key findings of this study is the strong association between living with children aged ≤ 6 years and the risk of pneumonia in older adults, consistent with previous reports that have identified regular contact with children or the number of children in the household as CAP risk factors in adults [8, 24, 25]. More recent data also suggest that contact with children may contribute to adult pneumococcal CAP, with one study reporting an increased incidence of adult pneumococcal CAP during school holiday periods [26]. However, evidence specifically focusing on older adults living with young children aged ≤ 6 years in the post-PCV13 era remains limited. In the national data, three-generation households accounted for only 11.0% of households including persons aged ≥ 65 years in 2016 and 9.4% in 2019 [27]. Although not directly equivalent to living with children aged ≤ 6 years, these data contextualize the relatively high proportion of older persons living with children aged ≤ 6 years observed among CAP cases in our study (15% vs. 3% in controls). Notably, this association was observed consistently across both age subgroups (65–74 years and ≥ 75 years) and remained significant in the subgroup analysis limited to pneumococcal pneumonia, despite the limited sample size. This finding suggests that the risk posed by contact with young children persists in the post-PCV13 era and is likely driven by mechanisms beyond direct pneumococcal transmission alone. In this study, individual-level PCV13 vaccination histories of children living in the participants’ households were unavailable; however, WHO and United Nations Children’s Fund estimates showed very high national PCV13 coverage in Japan during the study period: 99% in 2016, 99% in 2017, 98% in 2018, and 97% in 2019 [28]. Therefore, most young children were likely to have received the PCV13. Although non-vaccine serotypes or persistent vaccine serotypes (e.g., serotype 3, 19 A) of *Streptococcus pneumoniae* may still circulate [15], the transmission of respiratory viruses from children to older adults likely plays a pivotal role [29]. Children frequently serve as reservoirs for respiratory viruses such as respiratory syncytial virus, influenza, and rhinovirus [29, 30]. Transmission of these viruses to older household members can damage the respiratory mucosal barrier and impair mucociliary clearance [31, 32]. This virus-induced damage modulates local immune responses and significantly increases susceptibility to secondary bacterial superinfections, specifically pneumococcal pneumonia [33]. Thus, even if vaccine-type pediatric pneumococcal carriage has decreased due to vaccination with PCV13, the transmission of respiratory viruses by children may continue to drive the incidence of pneumococcal pneumonia in older adults through viral-bacterial synergism. Our findings highlight the important role of intergenerational transmission of infectious pathogens even in the post-PCV13 era, indicating an ongoing need for prevention strategies in households with children and older adults.

Impaired ADL status was also independently associated with an increased risk of pneumonia. This observation aligns with recent systematic reviews demonstrating that functional impairment—such as low or intermediate levels of dependence (defined as a Barthel Index < 100), incontinence, mobility limitations, and being bedridden—is a risk factor for pneumonia [9]. Potential explanations include reduced physical activity leading to respiratory muscle weakness, impaired airway clearance, and decreased pulmonary ventilation efficiency [34]. Moreover, subtle, unnoticed micro-aspiration events, facilitated by weakened pharyngeal musculature and poor oral hygiene, may alter pulmonary microbiota, enhancing vulnerability to pneumonia [35, 36]. Although clinically diagnosed aspiration pneumonia (e.g., caused by inhalation during eating or vomiting) was among the exclusion criteria in this study, undiagnosed, subclinical micro-aspiration might have contributed to the elevated CAP risk observed among individuals with impaired ADL [37]. Therefore, our study reinforces the importance of maintaining functional independence and implementing interventions such as swallowing rehabilitation and good oral hygiene to reduce aspiration-related pneumonia among older adults.

Our results further revealed that a BMI below 18.5 kg/m² was associated with a significantly increased risk of pneumonia in older adults. Similar to previous findings, low BMI may reflect inadequate nutrition and diminished immune function [38], as well as reduced respiratory muscle strength needed to clear secretions effectively [39]. Conversely, BMI ≥ 25.0 kg/m² was associated with lower odds of CAP in our analysis. Evidence regarding BMI and CAP incidence is mixed, and a meta-analysis suggests a J-shaped association, with increased risk among underweight individuals and little or no clear protection in obesity; overweight may be similar to, or slightly lower than, normal weight in the context of CAP risk [40]. Given that the BMI threshold of 25 kg/m² in Japan largely corresponds to “overweight” in many Western studies [41], our finding may reflect lower frailty and better nutritional reserve in mildly overweight older adults rather than a direct protective effect of adiposity. Importantly, several studies have reported an “obesity paradox” for outcomes among patients hospitalized with CAP (e.g., lower mortality in overweight/obese patients) [42], but these prognostic associations should not be conflated with the risk of developing CAP.

In the age-stratified analyses, respiratory comorbidities such as COPD and bronchial asthma were independently associated with CAP only among participants aged ≥ 75 years. This pattern is biologically plausible: immunosenescence and age-related inflammation intensify with advancing age, weakening mucosal and innate defenses and magnifying the impact of chronic airway inflammation on infection risk [43]. Specifically for COPD, older adults are likely to have more advanced disease with greater structural lung damage and reduced pulmonary reserve compared with that of younger adults, making them more susceptible to pneumonia progression. Moreover, asthma and severe COPD have been linked to an increased risk of pneumococcal disease and CAP in adults [9, 44]. The risk may be further influenced by long-term exposure to inhaled corticosteroids (ICS), often prescribed for asthma or severe COPD, which increase susceptibility to pneumonia in a dose-dependent manner [45]. Consequently, the interplay between advanced age, disease severity, and ICS use likely increases the risk associated with these respiratory conditions, specifically in the older age stratum. An alternative explanation is limited power and confounding in the younger age stratum, in which strong predictors such as low BMI and impaired ADL may have attenuated the associations with other respiratory conditions observed. These age-specific findings highlight the need to prioritize infection-prevention strategies in older adults with COPD and asthma [11], including vaccination, careful stewardship of high-dose ICS when feasible [45].

In the present study, the presence of gastrointestinal disease was associated with a reduced risk of CAP among participants aged 65–74 years. This finding contrasts with those of previous studies that have identified gastrointestinal disorders, particularly gastroesophageal reflux disease [46] and the use of gastric acid-suppressants [9] as risk factors for CAP. Because this inverse association was observed only in the 65–74-years age group and was not present in the overall analysis or among those aged ≥ 75 years, it may reflect differences in healthcare utilization patterns among hospital outpatients rather than a biological protective effect. Controls were recruited from outpatients visiting the same medical institutions as did the cases, predominantly from hospital-based internal medicine departments or clinics; thus, conditions requiring regular follow-up, including chronic gastrointestinal diseases, may have been overrepresented in the control group relative to their occurrence in the general population. Furthermore, the definition of gastrointestinal disease was based on self-reports, and data regarding specific diagnoses and medication use, including the use of gastric acid-suppressants, were not available. Consequently, this variable does not necessarily reflect exposure to potent gastric acid-suppressants, which is a known risk factor for CAP [9]. Therefore, this finding should be considered exploratory and interpreted with caution.

This study highlights critical points for clinical and public health strategies. CAP prevention should be guided by age-specific dominant risk factors. Nutritional screening and intervention programs for undernourished older adults may be important, given their potential to enhance immunity and reduce pneumonia risk. Infection-control measures within intergenerational households—such as hand hygiene, respiratory etiquette, and isolation strategies when young children exhibit respiratory signs—may help reduce transmission of respiratory pathogens [47, 48], although direct evidence for CAP prevention in older adults remains limited. For adults aged ≥ 75 years in particular, a greater emphasis should be placed on COPD and asthma management and to strategies for maintaining functional status or reducing aspiration-related risks. Although pneumococcal and influenza vaccination were not significantly associated with all-cause CAP or pneumococcal CAP in the adjusted models in this study, this secondary analysis was designed to identify clinical and lifestyle-related risk factors rather than to evaluate vaccine effectiveness; therefore, these vaccination-related estimates should be interpreted cautiously. As discussed in detail in the original study [7], potential reasons for the lack of detectable vaccine effectiveness include indirect effects of routine childhood PCV13 vaccination—namely herd immunity and serotype replacement of *Streptococcus pneumoniae*, with non-vaccine serotypes reportedly increasing from 28% to 49% in Japan after childhood PCV13 introduction [15] — as well as methodological differences from earlier studies and unmeasured confounding. Finally, continued surveillance of pneumococcal serotypes and the promotion of appropriate adult pneumococcal vaccination strategies remain essential to minimize risks from *Streptococcus pneumoniae* serotypes not covered by childhood PCV13.

This study has several limitations. First, although we have adjusted for known confounders, caution must be exercised when making causal inferences owing to the potential for reverse causality. For example, the observed association with low BMI might reflect weight loss resulting from pre-existing frailty or subclinical conditions rather than being a primary cause of pneumonia. Additionally, the reliance on self-administered questionnaires for vaccination history and lifestyle factors may have introduced recall bias. In addition, respiratory comorbidities such as COPD and asthma were ascertained from questionnaire-reported physician diagnoses. Because COPD is frequently underdiagnosed in the general population [49], this approach may have led to under-ascertainment of undiagnosed COPD. Indeed, the observed COPD prevalence in our study was lower than that reported in spirometry-based studies of older Japanese adults [49]. This potential exposure misclassification should be considered when interpreting the associations between respiratory comorbidities and CAP. Second, the sample size of 142 cases and 596 controls might not have provided sufficient statistical power to identify some associations. For example, COPD was not significantly associated with CAP in the overall analysis, with the lower limit of the 95% CI falling slightly below 1.0 (adjusted OR 1.69, 95% CI 0.92–3.08), although its association with CAP was statistically significant in the subgroup aged ≥ 75 years. This lack of a statistically significant association between COPD and CAP might be attributable to limited statistical power. In addition, the number of pneumococcal CAP cases was limited (*n* = 31), which precluded reliable age-stratified multivariable analyses; therefore, the findings from the pneumococcal CAP analysis should be interpreted with caution. Third, sociodemographic factors such as educational level and economic status were not assessed and could be a source of unmeasured confounding. Fourth, biochemical markers of nutritional status, such as total protein, serum albumin, and hemoglobin, were not available for this analysis. The reliance on BMI as the sole indicator of nutritional status is a limitation. Fifth, the generalizability of our findings may be limited, because the research was conducted solely on data from Japan, and the results may not be generalizable to other countries with different healthcare systems and population characteristics. Moreover, the original study was designed to evaluate the effectiveness of PPSV23 against CAP defined according to the guidelines applicable during the study period. This specific focus and definition led to the exclusion of immunocompromised patients and those with malignant tumors. This, in turn, limits the applicability of our findings to the broader older adult population, particularly those at high risk who were specifically excluded in this study. Future research with more detailed etiological analyses would be beneficial in elucidating the precise pathways that connect the identified risk factors to pneumonia in older adults.

## Conclusions

In conclusion, even after the introduction of childhood PCV13, living with children aged ≤ 6 years, low BMI, and impaired ADL remain major risk factors for CAP among older adults in Japan. Importantly, our age-stratified analysis revealed distinct risk profiles: although intergenerational transmission was a predominant risk regardless of age, respiratory comorbidities including COPD and asthma and impaired ADL emerged as significant independent risks in individuals aged ≥ 75 years. Conversely, a higher BMI was associated with a reduced risk. These findings suggest that pneumonia prevention in older adults should take age-specific dominant risk factors into account. Infection-control measures in households with young children and nutritional support may be relevant across older age groups, whereas careful management of COPD and asthma and strategies to maintain functional status or reduce aspiration-related risks may be particularly important among adults aged ≥ 75 years.

## Supplementary Information

Below is the link to the electronic supplementary material.

Supplementary Material 1

Supplementary Material 2

## Data Availability

All datasets generated and analyzed during the study are available from the corresponding author on reasonable request.

## Abbreviations

ADL: Activities of daily living
aOR: Adjusted odds ratio
BMI: Body mass index
CAP: Community-acquired pneumonia
CI: Confidence interval
COPD: Chronic obstructive pulmonary disease
OR: Odds ratio
PCV7: Pneumococcal conjugate vaccine, 7-valent
PCV13: Pneumococcal conjugate vaccine, 13-valent
PPSV23: Pneumococcal polysaccharide vaccine, 23-valent
WHO: World Health Organization
