# Supplementary material for: Risk factors for community-acquired pneumonia in older adults in Japan after the introduction of the childhood PCV13
Source: BMC Infect Dis. 2026 Jun 16;26:1557. doi: 10.1186/s12879-026-13820-7 (PMC13505070; doi:10.1186/s12879-026-13820-7)
Supplement: Supplementary file 1 [file 12879_2026_13820_MOESM1_ESM.docx]

**Supplementary Material 1**

**English-Language Version of the Study Questionnaires**

This supplementary material contains English translations of the two questionnaires used in the original study (Nakashima K et al., Vaccine, 2022). Both questionnaires were developed specifically for that study and have not been published elsewhere. The original questionnaires were administered in Japanese.

Part A: Physician-Administered Questionnaire (pp. 2–3)

Part B: Self-Administered Questionnaire on Lifestyle and Living Environment (pp. 4–6)

ID Number : ____________________

**Survey on the Preventive Effects of Influenza Vaccine and Pneumococcal Vaccine against Pneumonia**

**Part A: Physician-Administered Questionnaire**

**Exclusion criteria (for both cases and controls):**

(1) Obvious aspiration or vomiting, (2) Malignant tumor, (3) Currently receiving oral corticosteroids / immunosuppressants, (4) History of splenectomy

Date of enrollment request: _____ / _____ / _____ (YYYY/MM/DD)

Physician name: ______________________________ Medical institution: ______________________________

Case/Control status: 1. Case 2. Control (control for case name: ______________________ ), Disease name: ______________________

Department: 1. Internal medicine	2. Other ( ______________________ )	3. Clinic

**Section 1: Patient Background (For Both Cases and Controls)**

| **Name** | Chart No.: _______________ |
| --- | --- |
| **Date of birth** | _____ / _____ / _____ (YYYY/MM/DD) Age: ______ years Sex: Male / Female |
| **Underlying**  **respiratory**  **diseases** | 1. Present 2. Absent  If present, check all that apply:  □ Chronic obstructive pulmonary disease (COPD)  (pulmonary emphysema / chronic bronchitis)  □ Interstitial pneumonia  □ Bronchial asthma  □ Pulmonary tuberculosis (sequelae)  □ Other respiratory disease  (Disease name: ___________________________________ ) |
| **Blood tests** | 1. Not performed  2. Performed (Date: _____ / _____ / _____ YYYY/MM/DD)  Total protein: _______ g/dL  Serum albumin: _______ g/dL  Hemoglobin: _______ g/dL |
| **Pneumococcal**  **vaccine** | 1. Vaccinated 2. Not vaccinated 3. Unknown  If vaccinated:  PPSV23, 1st dose (Date: _____ / _____ YYYY/MM)  PPSV23, 2nd dose (Date: _____ / _____ YYYY/MM)  PCV13 (Date: _____ / _____ YYYY/MM) |
| **Influenza**  **vaccine** | 1. Vaccinated (Date: _____ / _____ YYYY/MM)  2. Not vaccinated  3. Unknown |

**Section 2: Pneumonia-Related Clinical Information (For Cases Only)**

| **Date of**  **pneumonia**  **diagnosis** | _____ / _____ / _____ (YYYY/MM/DD) |
| --- | --- |
| **Fever**  **(≥37.5°C)** | 1. Present 2. Absent 3. Unknown |
| **Cough** | 1. Present 2. Absent 3. Unknown |
| **Sputum** | 1. Present 2. Absent 3. Unknown |
| **Influenza**  **rapid diagnostic**  **test** | 1. Not performed  2. Performed (Date: _____ / _____ / _____ YYYY/MM/DD)  Result: Positive / Negative |
| **Pneumococcal**  **urinary antigen**  **test** | 1. Not performed  2. Performed (Date: _____ / _____ / _____ YYYY/MM/DD)  Result: Positive / Negative |
| **Sputum culture** | 1. Not performed  2. Performed (Date: _____ / _____ / _____ YYYY/MM/DD)  Streptococcus pneumoniae detected: 1. Yes 2. No |
| **Clinical course** | Maximum body temperature: _______ °C  Maximum white blood cell count: _______ /μL  Maximum CRP: _______ mg/dL |
| **Outcome** | Date: _____ / _____ / _____ (YYYY/MM/DD)  1. Cured 2. Improved 3. Worsened 4. Died 5. Unknown |

ID Number: ____________________

**Survey on the Preventive Effects of Influenza Vaccine and Pneumococcal Vaccine against Pneumonia**

**Part B: Self-Administered Questionnaire on Lifestyle and Living Environment**

Secretariat: Kei Nakashima, Director, Department of Pulmonology, Kameda Medical Center

929 Higashi-cho, Kamogawa, Chiba 296-0041, Japan

Tel: 04-7092-2211	Fax: 04-7099-1198	E-mail: kei.7.nakashima@gmail.com

Date of completion: _____ / _____ / _____ (YYYY/MM/DD)

Patient name: ___________________________________	Sex: 1. Male	2. Female

Date of birth: _____ / _____ / _____ (YYYY/MM/DD)	Age: ______ years

Address: Prefecture ________________ City ________________ Ward/District ________________

Person who completed this form:

1. The patient (self)

2. Someone other than the patient → Name: __________________________ Relationship to the patient: ______________

Contact in case of missing information:

Name: __________________________	Phone number: __________________________

**Question 1.** Please answer the following about the patient.

Height: _______ . _______ cm	Weight: _______ . _______ kg

**Question 2.** Where does the patient primarily live?

1. At home	2. Senior housing/apartment (without long-term care insurance)

◆ Number of household members (including the patient): ( _______ ) persons

◆ Does the patient live with any children aged 6 years or younger?

1. Yes	2. No

**Question 3.** Does the patient currently have a spouse?

1. Yes (including common-law partner) → 1. Living together	2. Living separately

2. No → 1. Divorced	2. Widowed	3. Never married

**Question 4.** Which of the following best describes the patient’s daily life? Please circle one number.

| 1. | Spends the entire day in bed. | Cannot turn over by oneself. |
| --- | --- | --- |
| 2. | Spends the entire day in bed. | Can turn over and change position by oneself. |
| 3. | Requires partial assistance from a caregiver for eating, toileting, or dressing; spends most of the day in bed. | Requires assistance to transfer to a wheelchair, chair, or portable toilet. |
| 4. | Requires partial assistance from a caregiver for eating, toileting, or dressing; spends most of the day in bed. | Can transfer to a wheelchair, chair, or portable toilet without assistance. |
| 5. | Can eat, use the toilet, and dress mostly independently; spends most of the day out of bed. | Rarely goes out, even with a caregiver available. |
| 6. | Can eat, use the toilet, and dress mostly independently; spends most of the day out of bed. | Goes out with assistance. |
| 7. | Can manage personal daily activities mostly independently. | Goes out alone to nearby places. |
| 8. | Can manage personal daily activities mostly independently. | Goes out alone using public transportation. |

**Question 5.** Has the patient been diagnosed by a physician with any of the following conditions? Please circle all that apply.

1. Hypertension	2. Dyslipidemia	3. Heart disease

4. Cerebral hemorrhage / cerebral infarction / stroke	5. Diabetes mellitus	6. Kidney disease

7. Liver disease	8. Gastrointestinal disease

9. Other ( _________________________________ )

0. None

**Question 6.** Is the patient currently receiving home oxygen therapy?

1. Yes	2. No

**Question 7.** Has the patient ever been diagnosed by a physician with any of the following respiratory diseases?

◆ Chronic obstructive pulmonary disease (COPD) (pulmonary emphysema / chronic bronchitis): 1. Yes	2. No

◆ Interstitial pneumonia: 1. Yes	2. No

◆ Bronchial asthma: 1. Yes	2. No

◆ Pulmonary tuberculosis (sequelae): 1. Yes	2. No

◆ Other respiratory disease: 1. Yes	2. No	(Disease name: _________________________ )

**Question 8.** Has the patient ever received a pneumococcal vaccine?

1. Yes	2. No	3. Don’t know

**If yes:**

◆ When was the vaccination administered?

1. Within the past year	2. ( _______ ) years ago	3. Unknown

◆ Where was the vaccination administered?

Name of medical institution: ( _________________________________ )

**Question 9.** Has the patient received an influenza vaccine within the past 6 months?

1. Yes	2. No	3. Don’t know

**Question 10.** How many times has the patient received an influenza vaccine in the past 3 years in total?

Total: ( _______ ) times

**Question 11.** Does the patient currently smoke?

1. No, never smoked

2. Used to smoke but quit ( _______ ) years ago

3. Yes, currently smokes

**Question 12.** Does the patient currently drink alcohol?

1. No, never drank

2. Used to drink but quit

3. Drinks occasionally

4. Drinks daily

◆ For each of the following beverages, please indicate the frequency and amount consumed in the past month.

**Question 13.** Coffee

1. Did not drink

2. ( _______ ) cups per month

3. ( _______ ) cups per week

4. ( _______ ) cups per day

**Question 14.** Black tea

1. Did not drink

2. ( _______ ) cups per month

3. ( _______ ) cups per week

4. ( _______ ) cups per day

**Question 15.** Green tea

1. Did not drink

2. ( _______ ) cups per month

3. ( _______ ) cups per week

4. ( _______ ) cups per day

**This is the end of the questionnaire. Thank you very much for your cooperation.**
