# Supplementary material for: Risk factors for community-acquired pneumonia in older adults in Japan after the introduction of the childhood PCV13
Source: BMC Infect Dis. 2026 Jun 16;26:1557. doi: 10.1186/s12879-026-13820-7 (PMC13505070; doi:10.1186/s12879-026-13820-7)
Supplement: Supplementary file 2 [file 12879_2026_13820_MOESM2_ESM.docx]

**Risk factors for community-acquired pneumonia in older adults in Japan after the introduction of the childhood PCV13**

**Supplementary Material 2**

Supplementary Table 1. Baseline characteristics of participants aged 65–74 years (N = 390)

| Characteristics | Cases  (N = 74) | Controls  (N = 316) | p ^b^ |
| --- | --- | --- | --- |
| Age ^a^ |  |  |  |
| Median (IQR) | 70 (68–73) | 71 (68–73) | 0.775 |
| Sex |  |  |  |
| Male | 47 (64%) | 197 (62%) | 0.851 |
| Female | 27 (36%) | 119 (38%) |  |
| Vaccination |  |  |  |
| Pneumococcal vaccine | 42 (57%) | 167 (53%) | 0.544 |
| PPSV23 only | 41 (55%) | 157 (50%) |  |
| PCV13 only | 0 (0%) | 2 (1%) |  |
| Both | 0 (0%) | 6 (2%) |  |
| unknown | 1 (1%) | 2 (1%) |  |
| Influenza vaccine | 31 (42%) | 129 (41%) | 0.866 |
| Respiratory diseases |  |  |  |
| COPD | 18 (24%) | 48 (15%) | 0.059 |
| Interstitial pneumonia | 4 (5%) | 15 (5%) | 0.767 |
| Bronchial asthma | 10 (14%) | 44 (14%) | 0.927 |
| Tuberculosis (sequelae) | 1 (1%) | 3 (1%) | 0.571 |
| Underlying diseases |  |  |  |
| Hypertension | 29 (39%) | 144 (46%) | 0.320 |
| Dyslipidemia | 13 (18%) | 93 (29%) | 0.039 |
| Diabetes | 16 (22%) | 81 (26%) | 0.472 |
| Chronic kidney disease | 4 (5%) | 28 (9%) | 0.330 |
| Heart disease | 10 (14%) | 50 (16%) | 0.620 |
| Stroke | 2 (3%) | 6 (2%) | 0.650 |
| Gastrointestinal disease | 3 (4%) | 37 (12%) | 0.051 |
| Liver disease | 1 (1%) | 18 (6%) | 0.143 |
| BMI (kg/m²) |  |  |  |
| <18.5 | 16 (22%) | 32 (10%) | 0.003 |
| 18.5–24.9 | 47 (64%) | 190 (60%) |  |
| ≥25.0 | 11 (15%) | 94 (30%) |  |
| Living with children aged ≤6 years |  |  |  |
| None | 61 (82%) | 305 (97%) | <0.001 |
| Yes | 13 (18%) | 11 (3%) |  |
| Activities of daily living |  |  |  |
| Independent | 70 (95%) | 310 (98%) | 0.101 |
| Not independent | 4 (5%) | 6 (2%) |  |
| Alcohol use history |  |  |  |
| Never/past | 41 (55%) | 178 (56%) | 0.740 |
| Occasionally | 17 (23%) | 61 (19%) |  |
| Daily | 16 (22%) | 77 (24%) |  |
| Smoking history |  |  |  |
| Never smoker | 32 (43%) | 153 (48%) | 0.669 |
| Past smoker | 31 (42%) | 125 (40%) |  |
| Current smoker | 11 (15%) | 38 (12%) |  |

^a^ Age indicates chronological age at the time of diagnosis or enrollment.

^b^ The Wilcoxon rank-sum test, chi-square test, or Fisher’s exact test was performed as appropriate.

BMI, body mass index, COPD, chronic obstructive pulmonary disease; IQR, interquartile range; PCV13, 13-valent pneumococcal conjugate vaccine; PPSV23, 23-valent pneumococcal polysaccharide vaccine.

Supplementary Table 2. Baseline characteristics of participants aged ≥75 years (N = 348)

| Characteristics | Cases  (N = 68) | Controls  (N = 280) | p ^b^ |
| --- | --- | --- | --- |
| Age ^a^, median (IQR) | 81 (78–83) | 81 (78–83) | 0.807 |
| Sex |  |  |  |
| Male | 32 (47%) | 118 (42%) | 0.463 |
| Female | 36 (53%) | 162 (58%) |  |
| Vaccination |  |  |  |
| Pneumococcal vaccine | 41 (60%) | 167 (60%) | 0.922 |
| PPSV23 only | 38 (56%) | 153 (55%) |  |
| PCV13 only | 1 (1%) | 7 (3%) |  |
| Both | 1 (1%) | 5 (2%) |  |
| unknown | 1 (1%) | 1 (0.4%) |  |
| Influenza vaccine | 28 (41%) | 133 (48%) | 0.348 |
| Respiratory diseases |  |  |  |
| COPD | 16 (24%) | 35 (13%) | 0.021 |
| Interstitial pneumonia | 2 (3%) | 9 (3%) | 1.000 |
| Bronchial asthma | 13 (19%) | 30 (11%) | 0.059 |
| Tuberculosis (sequelae) | 4 (6%) | 6 (2%) | 0.110 |
| Underlying diseases |  |  |  |
| Hypertension | 38 (56%) | 176 (63%) | 0.289 |
| Dyslipidemia | 14 (21%) | 81 (29%) | 0.166 |
| Diabetes | 19 (28%) | 57 (20%) | 0.175 |
| Chronic kidney disease | 5 (7%) | 26 (9%) | 0.616 |
| Heart disease | 9 (13%) | 53 (19%) | 0.271 |
| Stroke | 4 (6%) | 21 (8%) | 0.797 |
| Gastrointestinal disease | 10 (15%) | 47 (17%) | 0.678 |
| Liver disease | 2 (3%) | 10 (4%) | 1.000 |
| BMI (kg/m²) |  |  |  |
| <18.5 | 17 (25%) | 32 (11%) | 0.012 |
| 18.5–24.9 | 40 (59%) | 182 (65%) |  |
| ≥25.0 | 11 (16%) | 66 (24%) |  |
| Living with children aged ≤6 years |  |  |  |
| No | 60 (88%) | 275 (98%) | <0.001 |
| Yes | 8 (12%) | 5 (2%) |  |
| Activities of daily living |  |  |  |
| Independent | 58 (85%) | 259 (93%) | 0.061 |
| Not independent | 10 (15%) | 21 (8%) |  |
| Alcohol use history |  |  |  |
| Never/past | 47 (69%) | 196 (70%) | 0.985 |
| Occasionally | 10 (15%) | 41 (15%) |  |
| Daily | 11 (16%) | 43 (15%) |  |
| Smoking history |  |  |  |
| Never smoker | 43 (63%) | 190 (68%) | 0.070 |
| Past smoker | 18 (26%) | 80 (29%) |  |
| Current smoker | 7 (10%) | 10 (4%) |  |

^a^ Age indicates chronological age at the time of diagnosis or enrollment.

^b^ Wilcoxon rank-sum test, chi-square test, or Fisher’s exact test was performed as appropriate.

BMI, body mass index; COPD, chronic obstructive pulmonary disease; IQR, interquartile range; PCV13, 13-valent pneumococcal conjugate vaccine; PPSV23, 23-valent pneumococcal polysaccharide vaccine.

Supplementary Table 3. Baseline characteristics of participants in the sensitivity analysis using self-administered respiratory disease status (N = 686)

| Characteristics | Cases  (N = 133) | Controls  (N = 553) | p ^b^ |
| --- | --- | --- | --- |
| Age (years),^a^ median (IQR) | 75 (70–81) | 75 (71–81) | 0.878 |
| Sex |  |  |  |
| Male | 71 (53%) | 283 (51%) | 0.647 |
| Female | 62 (47%) | 270 (49%) |  |
| Vaccination |  |  |  |
| Pneumococcal vaccine | 79 (59%) | 300 (54%) | 0.283 |
| PPSV23 only | 75 (56%) | 279 (50%) |  |
| PCV13 only | 1 (1%) | 9 (2%) |  |
| Both | 1 (1%) | 10 (2%) |  |
| Unknown | 2 (1%) | 2 (0.4%) |  |
| Influenza vaccine | 56 (42%) | 241 (44%) | 0.758 |
| Respiratory diseases |  |  |  |
| COPD | 32 (24%) | 71 (13%) | 0.001 |
| Interstitial pneumonia | 3 (2%) | 18 (3%) | 0.780 |
| Bronchial asthma | 19 (14%) | 67 (12%) | 0.497 |
| Tuberculosis (sequelae) | 5 (4%) | 9 (2%) | 0.162 |
| Underlying diseases |  |  |  |
| Hypertension | 64 (48%) | 302 (54%) | 0.178 |
| Dyslipidemia | 25 (19%) | 170 (31%) | 0.006 |
| Diabetes | 33 (24%) | 128 (23%) | 0.684 |
| Chronic kidney disease | 8 (6%) | 54 (10%) | 0.178 |
| Heart disease | 17 (13%) | 96 (17%) | 0.201 |
| Stroke | 6 (5%) | 26 (5%) | 0.926 |
| Gastrointestinal disease | 13 (10%) | 80 (15%) | 0.156 |
| Liver disease | 3 (2%) | 27 (5%) | 0.183 |
| BMI (kg/m²) |  |  |  |
| <18.5 | 33 (25%) | 57 (10%) | <0.001 |
| 18.5-24.9 | 79 (59%) | 350 (63%) |  |
| ≥ 25.0 | 21 (16%) | 146 (26%) |  |
| Living with children aged ≤6 years |  |  |  |
| No | 113 (85%) | 540 (98%) | <0.001 |
| Yes | 20 (15%) | 13 (2%) |  |
| Activities of daily living |  |  |  |
| Independent | 120 (90%) | 526 (95%) | 0.031 |
| Not independent | 13 (10%) | 27 (5%) |  |
| Alcohol use history |  |  |  |
| Never/Past | 82 (62%) | 374 (63%) | 0.823 |
| Occasionally | 26 (20%) | 96 (17%) |  |
| Daily | 25 (19%) | 110 (20%) |  |
| Smoking history |  |  |  |
| Never smoker | 71 (53%) | 326 (59%) | 0.137 |
| Past smoker | 44 (33%) | 182 (33%) |  |
| Current smoker | 18 (14%) | 45 (8%) |  |

^a^ Age indicates chronological age at the time of diagnosis or enrollment.

^b^ The p values were calculated using the Wilcoxon rank-sum test, chi-square test, or Fisher’s exact test, as appropriate.

BMI, body mass index; COPD, chronic obstructive pulmonary disease; IQR, interquartile range; PCV13, 13-valent pneumococcal conjugate vaccine; PPSV23, 23-valent pneumococcal polysaccharide vaccine

Supplementary Table 4. Baseline characteristics of participants aged 65–74 years in the sensitivity analysis using self-administered respiratory disease status (N = 351)

| Characteristics | Cases  (N = 67) | Controls  (N = 284) | p ^b^ |
| --- | --- | --- | --- |
| Age ^a^ |  |  |  |
| Median (IQR) | 70 (67–73) | 71 (68–73) | 0.627 |
| Sex |  |  |  |
| Male | 40 (60%) | 170 (60%) | 0.981 |
| Female | 27 (40%) | 114 (40%) |  |
| Vaccination |  |  |  |
| Pneumococcal vaccine | 38 (57%) | 139 (49%) | 0.252 |
| PPSV23 only | 37 (57%) | 127 (45%) |  |
| PCV13 only | 0 (0%) | 6 (2%) |  |
| Both | 0 (0%) | 5 (2%) |  |
| unknown | 1 (1%) | 1 (0.4%) |  |
| Influenza vaccine | 28 (42%) | 113 (40%) | 0.763 |
| Respiratory diseases |  |  |  |
| COPD | 15 (22%) | 38 (13%) | 0.064 |
| Interstitial pneumonia | 2 (3%) | 9 (3%) | 1.000 |
| Bronchial asthma | 6 (9%) | 39 (13%) | 0.293 |
| Tuberculosis (sequelae) | 1 (1%) | 3 (1%) | 0.573 |
| Underlying diseases |  |  |  |
| Hypertension | 26 (39%) | 133 (47%) | 0.235 |
| Dyslipidemia | 11 (16%) | 89 (31%) | 0.015 |
| Diabetes | 15 (22%) | 74 (26%) | 0.534 |
| Chronic kidney disease | 3 (4%) | 28 (10%) | 0.163 |
| Heart disease | 8 (12%) | 44 (15%) | 0.462 |
| Stroke | 2 (3%) | 5 (2%) | 0.622 |
| Gastrointestinal disease | 3 (4%) | 34 (12%) | 0.072 |
| Liver disease | 1 (1%) | 17 (6%) | 0.215 |
| BMI (kg/m²) |  |  |  |
| <18.5 | 16 (24%) | 27 (10%) | 0.001 |
| 18.5–24.9 | 41 (61%) | 174 (61%) |  |
| ≥25.0 | 10 (15%) | 83 (29%) |  |
| Living with children aged ≤6 years |  |  |  |
| No | 55 (82%) | 276 (97%) | <0.001 |
| Yes | 12 (18%) | 8 (3%) |  |
| Activities of daily living |  |  |  |
| Independent | 64 (95%) | 278 (98%) | 0.381 |
| Not independent | 3 (5%) | 6 (2%) |  |
| Alcohol use history |  |  |  |
| Never/past | 37 (55%) | 158 (56%) | 0.623 |
| Occasionally | 16 (24%) | 55 (19%) |  |
| Daily | 14 (21%) | 71 (25%) |  |
| Smoking history |  |  |  |
| Never smoker | 29 (43%) | 142 (50%) | 0.524 |
| Past smoker | 27 (40%) | 107 (38%) |  |
| Current smoker | 11 (16%) | 35 (12%) |  |

^a^ Age indicates chronological age at the time of diagnosis or enrollment.

^b^ The Wilcoxon rank-sum test, chi-square test, or Fisher’s exact test was performed as appropriate.

BMI, body mass index, COPD, chronic obstructive pulmonary disease; IQR, interquartile range; PCV13, 13-valent pneumococcal conjugate vaccine; PPSV23, 23-valent pneumococcal polysaccharide vaccine

Supplementary Table 5. Baseline characteristics of participants aged ≥75 years in the sensitivity analysis using self-administered respiratory disease status (N = 335)

| Characteristics | Cases  (N = 66) | Controls  (N = 269) | p ^b^ |
| --- | --- | --- | --- |
| Age ^a^, median (IQR) | 81 (78–83) | 81 (78–83) | 0.807 |
| Sex |  |  |  |
| Male | 31 (47%) | 113 (42%) | 0.466 |
| Female | 35 (53%) | 156 (58%) |  |
| Vaccination |  |  |  |
| Pneumococcal vaccine | 41 (62%) | 161 (60%) | 0.736 |
| PPSV23 only | 38 (58%) | 147 (55%) |  |
| PCV13 only | 1 (2%) | 8 (3%) |  |
| Both | 1 (2%) | 5 (2%) |  |
| unknown | 1 (2%) | 1 (0.4%) |  |
| Influenza vaccine | 28 (42%) | 128 (48%) | 0.452 |
| Respiratory diseases |  |  |  |
| COPD | 17 (26%) | 33 (12%) | 0.006 |
| Interstitial pneumonia | 1 (2%) | 9 (3%) | 0.693 |
| Bronchial asthma | 13 (20%) | 28 (10%) | 0.039 |
| Tuberculosis (sequelae) | 4 (6%) | 6 (2%) | 0.113 |
| Underlying diseases |  |  |  |
| Hypertension | 38 (58%) | 169 (63%) | 0.432 |
| Dyslipidemia | 14 (21%) | 81 (30%) | 0.151 |
| Diabetes | 18 (27%) | 54 (20%) | 0.151 |
| Chronic kidney disease | 5 (8%) | 26 (10%) | 0.600 |
| Heart disease | 9 (14%) | 52 (19%) | 0.283 |
| Stroke | 4 (6%) | 21 (8%) | 0.796 |
| Gastrointestinal disease | 10 (15%) | 46 (17%) | 0.704 |
| Liver disease | 2 (3%) | 10 (4%) | 1.000 |
| BMI (kg/m²) |  |  |  |
| <18.5 | 17 (26%) | 30 (11%) | 0.008 |
| 18.5–24.9 | 38 (58%) | 176 (65%) |  |
| ≥25.0 | 11 (17%) | 63 (23%) |  |
| Living with children aged ≤6 years |  |  |  |
| No | 58 (88%) | 264 (98%) | <0.001 |
| Yes | 8 (12%) | 5 (2%) |  |
| Activities of daily living |  |  |  |
| Independent | 56 (85%) | 248 (92%) | 0.065 |
| Not independent | 10 (15%) | 21 (8%) |  |
| Alcohol use history |  |  |  |
| Never/past | 45 (68%) | 189 (70%) | 0.905 |
| Occasionally | 10 (15%) | 41 (15%) |  |
| Daily | 11 (17%) | 39 (15%) |  |
| Smoking history |  |  |  |
| Never smoker | 42 (64%) | 190 (68%) | 0.074 |
| Past smoker | 17 (26%) | 80 (28%) |  |
| Current smoker | 7 (11%) | 10 (4%) |  |

^a^ Age indicates chronological age at the time of diagnosis or enrollment.

^b^ The Wilcoxon rank-sum test, chi-square test, or Fisher’s exact test was performed as appropriate.

BMI, body mass index, COPD, chronic obstructive pulmonary disease; IQR, interquartile range; PCV13, 13-valent pneumococcal conjugate vaccine; PPSV23, 23-valent pneumococcal polysaccharide vaccine.

Supplementary Table 6. Crude and adjusted ORs for CAP in older adults, overall and stratified by age group, in the sensitivity analysis using self-administered respiratory disease status

|  | Overall (65 ≥ years) (N = 686) | |  | 65–74 years (N = 351) | |  | ≥ 75 years (N = 335) | |
| --- | --- | --- | --- | --- | --- | --- | --- | --- |
|  | Crude OR (95% CI) | Adjusted OR* (95% CI) |  | Crude OR (95% CI) | Adjusted OR* (95% CI) |  | Crude OR (95% CI) | Adjusted OR* (95% CI) |
| COPD |  |  |  |  |  |  |  |  |
| No | 1 | 1 |  | 1 | 1 |  | 1 | 1 |
| Yes | 2.48 (1.41–4.40) | 1.73 (0.90–3.34) |  | 1.91 (0.91–4.05) | 1.21 (0.47–3.08) |  | 3.52 (1.46–8.48) | 3.93 (1.36–11.31) |
| Bronchial asthma |  |  |  |  |  |  |  |  |
| No | 1 | 1 |  | 1 | 1 |  | 1 | 1 |
| Yes | 1.36 (0.77–2.40) | 1.38 (0.75–2.57) |  | 0.64 (0.25–1.63) | 0.57 (0.21–1.55) |  | 2.57 (1.20–5.51) | 3.61 (1.42–9.14) |
| Tuberculosis (sequelae) |  |  |  |  |  |  |  |  |
| No | 1 | 1 |  | 1 | 1 |  | 1 | 1 |
| Yes | 2.59 (0.83–8.08) | 2.11 (0.61–7.29) |  | 1.67 (0.17–16.02) | 1.88 (0.13–26.70) |  | 3.08 (0.80–11.83) | 2.27 (0.52–9.98) |
| Hypertension |  |  |  |  |  |  |  |  |
| No | 1 | 1 |  | 1 | 1 |  | 1 | 1 |
| Yes | 0.76 (0.51–1.13) | 0.89 (0.56–1.41) |  | 0.70 (0.39–1.23) | 0.79 (0.41–1.54) |  | 0.84 (0.47–1.51) | 1.03 (0.50–2.10) |
| Dyslipidemia |  |  |  |  |  |  |  |  |
| No | 1 | 1 |  | 1 | 1 |  | 1 | 1 |
| Yes | 0.53 (0.32–0.90) | 0.71 (0.41–1.25) |  | 0.43 (0.20–0.93) | 0.56 (0.24–1.27) |  | 0.66 (0.31–1.35) | 0.75 (0.32–1.77) |
| Diabetes |  |  |  |  |  |  |  |  |
| No | 1 | 1 |  | 1 | 1 |  | 1 | 1 |
| Yes | 1.06 (0.68–1.65) | 1.38 (0.84–2.27) |  | 0.83 (0.44–1.58) | 1.17 (0.55–2.51) |  | 1.35 (0.72–2.54) | 1.92 (0.91–4.05) |
| Gastrointestinal disease |  |  |  |  |  |  |  |  |
| No | 1 | 1 |  | 1 | 1 |  | 1 | 1 |
| Yes | 0.62 (0.32–1.19) | 0.61 (0.30–1.25) |  | 0.29 (0.08–1.07) | 0.19 (0.04–0.89) |  | 0.88 (0.40–1.91) | 1.17 (0.47–2.87) |
| Liver disease |  |  |  |  |  |  |  |  |
| No | 1 | 1 |  | 1 | 1 |  | 1 | 1 |
| Yes | 0.42 (0.12–1.47) | 0.40 (0.11–1.52) |  | 0.22 (0.03–1.79) | 0.15 (0.02–1.43) |  | 0.74 (0.15–3.66) | 0.96 (0.17–5.31) |
| Chronic kidney disease |  |  |  |  |  |  |  |  |
| No | 1 | 1 |  | 1 | 1 |  | 1 | 1 |
| Yes | 0.61 (0.27–1.41) | 0.69 (0.28–1.72) |  | 0.41 (0.11–1.51) | 0.50 (0.12–2.01) |  | 0.87 (0.29–2.59) | 1.10 (0.31 –3.95) |
| BMI (kg/m²) |  |  |  |  |  |  |  |  |
| <18.5 | 2.43 (1.47–4.05) | 1.98 (1.11–3.51) |  | 2.22 (1.06–4.66) | 1.87 (0.77–4.56) |  | 2.64 (1.31–5.31) | 2.16 (0.93–5.05) |
| 18.5-24.9 | 1 | 1 |  | 1 | 1 |  | 1 | 1 |
| ≥25.0 | 0.62 (0.36–1.06) | 0.61 (0.34–1.08) |  | 0.50 (0.24–1.05) | 0.48 (0.21–1.08) |  | 0.79 (0.37–1.71) | 0.66 (0.28–1.57) |
| Living with children aged ≤6 years |  |  |  |  |  |  |  |  |
| No | 1 | 1 |  | 1 | 1 |  | 1 | 1 |
| Yes | 7.36 (3.49–15.52) | 6.73 (3.02–14.97) |  | 7.78 (2.87–21.07) | 5.23 (1.82–15.03) |  | 6.85 (2.22–21.14) | 13.14 (3.44–50.20) |
| Activities of daily living |  |  |  |  |  |  |  |  |
| Independent | 1 | 1 |  | 1 | 1 |  | 1 | 1 |
| Not independent | 1.91 (0.92–3.97) | 2.22 (0.99–4.98) |  | 1.88 (0.45–7.88) | 2.20 (0.32–15.34) |  | 1.92 (0.82–4.49) | 3.04 (1.13–8.17) |
| Smoking history |  |  |  |  |  |  |  |  |
| Never smoker | 1 | 1 |  | 1 | 1 |  | 1 | 1 |
| Past smoker | 1.32 (0.76–2.29) | 1.18 (0.63–2.21) |  | 1.49 (0.72–3.08) | 1.41 (0.62–3.23) |  | 1.12 (0.47–2.63) | 0.74 (0.24–2.27) |
| Current smoker | 2.23 (1.08–4.63) | 1.66 (0.74–3.76) |  | 1.83 (0.72–4.68) | 1.20 (0.42–3.46) |  | 3.16 (0.99–10.10) | 1.97 (0.48–8.11) |
| Pneumococcal vaccine |  |  |  |  |  |  |  |  |
| No | 1 | 1 |  | 1 | 1 |  | 1 | 1 |
| Yes | 1.40 (0.92–2.13) | 1.46 (0.91–2.36) |  | 1.58 (0.87–2.85) | 1.69 (0.83–3.44) |  | 1.25 (0.69–2.25) | 1.22 (0.60–2.48) |
| Influenza vaccine |  |  |  |  |  |  |  |  |
| No | 1 | 1 |  | 1 | 1 |  | 1 | 1 |
| Yes | 0.89 (0.56–1.43) | 0.84 (0.49–1.44) |  | 1.07 (0.56–2.05) | 0.93 (0.43–2.01) |  | 0.73 (0.37–1.45) | 0.79 (0.36–1.75) |

*The model incorporated all variables listed in the Table.

BMI, body mass index; CAP, community-acquired pneumonia; CI, confidence interval; COPD, chronic obstructive pulmonary disease; OR, odds ratio

Supplementary Table 7. Baseline characteristics of participants in the analysis limited to cases with pneumococcal CAP and their matched controls (N = 174)

| Characteristics | Cases  (N = 31) | Controls  (N = 143) | p ^b^ |
| --- | --- | --- | --- |
| Age ^a^, median (IQR) | 77 (68–80) | 76 (68–80) | 0.936 |
| Sex |  |  |  |
| Male | 13 (42%) | 57 (40%) | 0.831 |
| Female | 18 (58%) | 86 (60%) |  |
| Vaccination |  |  |  |
| Pneumococcal vaccine | 15 (48%) | 75 (53%) | 0.681 |
| PPSV23 only | 15 (48%) | 61 (43%) |  |
| PCV13 only | 0 (0%) | 7 (5%) |  |
| Both | 0 (0%) | 5 (4%) |  |
| unknown | 0 (0%) | 2 (1%) |  |
| Influenza vaccine | 11 (35%) | 54 (38%) | 0.812 |
| Respiratory diseases |  |  |  |
| COPD | 2 (6%) | 8 (6%) | 1.000 |
| Interstitial pneumonia | 2 (6%) | 5 (4%) | 0.610 |
| Bronchial asthma | 7 (23%) | 18 (13%) | 0.162 |
| Tuberculosis (sequelae) | 2 (6%) | 2 (1%) | 0.146 |
| Underlying diseases |  |  |  |
| Hypertension | 16 (52%) | 83 (58%) | 0.512 |
| Dyslipidemia | 4 (13%) | 37 (26%) | 0.123 |
| Diabetes | 8 (26%) | 39 (27%) | 0.868 |
| Chronic kidney disease | 1 (3%) | 10 (7%) | 0.691 |
| Heart disease | 2 (6%) | 19 (13%) | 0.376 |
| Stroke | 0 (0%) | 8 (6%) | 0.353 |
| Gastrointestinal disease | 0 (0%) | 9 (6%) | 0.365 |
| Liver disease | 0 (0%) | 4 (3%) | 1.000 |
| BMI (kg/m²) |  |  |  |
| <18.5 | 5 (16%) | 15 (10%) | 0.187 |
| 18.5–24.9 | 22 (71%) | 88 (62%) |  |
| ≥25.0 | 4 (13%) | 40 (28%) |  |
| Living with children (≤6 years) |  |  |  |
| No | 26 (84%) | 140 (98%) | 0.005 |
| Yes | 5 (16%) | 3 (2%) |  |
| Activities of daily living |  |  |  |
| Independent | 29 (94%) | 136 (95%) | 0.663 |
| Not independent | 2 (6%) | 7 (5%) |  |
| Alcohol use history |  |  |  |
| Never/past | 20 (65%) | 96 (67%) | 0.946 |
| Occasionally | 5 (16%) | 20 (14%) |  |
| Daily | 6 (19%) | 27 (19%) |  |
| Smoking history |  |  |  |
| Never smoker | 20 (65%) | 104 (73%) | 0.257 |
| Past smoker | 6 (19%) | 29 (20%) |  |
| Current smoker | 5 (16%) | 10 (7%) |  |

^a^ Age indicates chronological age at the time of diagnosis or enrollment.

^b^ Wilcoxon rank-sum test, chi-square test, or Fisher’s exact test was performed as appropriate.

BMI, body mass index; CAP, community-acquired pneumonia; COPD, chronic obstructive pulmonary disease; IQR, interquartile range; PCV13, 13-valent pneumococcal conjugate vaccine; PPSV23, 23-valent pneumococcal polysaccharide vaccine.

Supplementary Table 8. Baseline characteristics of participants in the analysis limited to pneumococcal CAP cases and their matched controls, in the sensitivity analysis using self-administered respiratory disease status (N = 159)

| Characteristics | Cases  (N = 29) | Controls  (N = 130) | p ^b^ |
| --- | --- | --- | --- |
| Age ^a^, median (IQR) | 77 (68–80) | 77 (68–80) | 0.968 |
| Sex |  |  |  |
| Male | 12 (41%) | 57 (38%) | 0.771 |
| Female | 17 (59%) | 86 (62%) |  |
| Vaccination |  |  |  |
| Pneumococcal vaccine | 15 (52%) | 65 (50%) | 0.867 |
| PPSV23 only | 15 (48%) | 50 (38%) |  |
| PCV13 only | 0 (0%) | 10 (8%) |  |
| Both | 0 (0%) | 4 (3%) |  |
| unknown | 0 (0%) | 1 (1%) |  |
| Influenza vaccine | 11 (38%) | 49 (38%) | 0.981 |
| Respiratory diseases |  |  |  |
| COPD | 4 (14%) | 4 (3%) | 0.037 |
| Interstitial pneumonia | 0 (0%) | 4 (3%) | 1.000 |
| Bronchial asthma | 7 (24%) | 16 (12%) | 0.140 |
| Tuberculosis (sequelae) | 2 (7%) | 2 (2%) | 0.152 |
| Underlying diseases |  |  |  |
| Hypertension | 15 (52%) | 77 (59%) | 0.459 |
| Dyslipidemia | 3 (10%) | 37 (28%) | 0.042 |
| Diabetes | 8 (28%) | 35 (27%) | 0.942 |
| Chronic kidney disease | 1 (3%) | 10 (8%) | 0.691 |
| Heart disease | 2 (7%) | 16 (12%) | 0.406 |
| Stroke | 0 (0%) | 8 (6%) | 0.353 |
| Gastrointestinal disease | 0 (0%) | 8 (6%) | 0.170 |
| Liver disease | 0 (0%) | 4 (3%) | 1.000 |
| BMI (kg/m²) |  |  |  |
| <18.5 | 5 (17%) | 14 (11%) | 0.239 |
| 18.5–24.9 | 20 (69%) | 80 (62%) |  |
| ≥25.0 | 4 (14%) | 36 (28%) |  |
| Living with children (≤6 years) |  |  |  |
| No | 24 (83%) | 127 (98%) | 0.006 |
| Yes | 5 (17%) | 3 (2%) |  |
| Activities of daily living |  |  |  |
| Independent | 27 (93%) | 123 (95%) | 0.669 |
| Not independent | 2 (7%) | 7 (5%) |  |
| Alcohol use history |  |  |  |
| Never/past | 19 (66%) | 87 (67%) | 0.884 |
| Occasionally | 5 (17%) | 18 (14%) |  |
| Daily | 5 (17%) | 25 (19%) |  |
| Smoking history |  |  |  |
| Never smoker | 19 (66%) | 97 (74%) | 0.278 |
| Past smoker | 5 (17%) | 23 (18%) |  |
| Current smoker | 5 (17%) | 10 (8%) |  |

^a^ Age indicates chronological age at the time of diagnosis or enrollment.

^b^ Wilcoxon rank-sum test, chi-square test, or Fisher’s exact test was performed as appropriate.

BMI, body mass index; COPD, chronic obstructive pulmonary disease; IQR, interquartile range; PCV13, 13-valent pneumococcal conjugate vaccine; PPSV23, 23-valent pneumococcal polysaccharide vaccine.

Supplementary Table 9. Crude and adjusted ORs for pneumococcal CAP in the sensitivity analysis using self-administered respiratory disease status (N = 159)

|  | Crude OR (95% CI) | Adjusted OR*  (95% CI) |
| --- | --- | --- |
| COPD |  |  |
| No | 1 | 1 |
| Yes | 5.17 (1.11–24.04) | 3.16 (0.44–22.70) |
| Bronchial asthma |  |  |
| No | 1 | 1 |
| Yes | 2.14 (0.77–5.94) | 3.19 (0.90–11.27) |
| Tuberculosis (sequelae) |  |  |
| No | 1 | 1 |
| Yes | 6.53 (0.56–76.72) | 3.07 (0.19–49.61) |
| Hypertension |  |  |
| No | 1 | 1 |
| Yes | 0.63 (0.25–1.56) | 0.72 (0.24–2.16) |
| Dyslipidemia |  |  |
| No | 1 | 1 |
| Yes | 0.26 (0.07–0.97) | 0.21 (0.04–1.03) |
| Diabetes |  |  |
| No | 1 | 1 |
| Yes | 1.00 (0.42–2.40) | 1.14 (0.36–3.62) |
| Chronic kidney disease |  |  |
| No | 1 | 1 |
| Yes | 0.40 (0.05–3.39) | 0.49 (0.04–5.47) |
| BMI (kg/m²) |  |  |
| <18.5 | 1.25 (0.39–4.01) | 1.40 (0.32–6.00) |
| 18.5–24.9 | 1 | 1 |
| ≥25.0 | 0.46 (0.15–1.43) | 0.75 (0.21–2.64) |
| Living with children aged ≤6 years |  |  |
| No | 1 | 1 |
| Yes | 10.46 (2.00–54.77) | 7.98 (1.30–48.90) |
| Activities of daily living |  |  |
| Independent | 1 | 1 |
| Not independent | 1.29 (0.26–6.50) | 3.48 (0.53–22.97) |
| Smoking history |  |  |
| Never smoker | 1 | 1 |
| Past smoker | 1.23 (0.33–4.63) | 0.88 (0.15–5.16) |
| Current smoker | 2.90 (0.75–11.14) | 1.58 (0.30–8.23) |
| Pneumococcal vaccine |  |  |
| No | 1 | 1 |
| Yes | 1.09 (0.47–2.56) | 1.37 (0.43–4.37) |
| Influenza vaccine |  |  |
| No | 1 | 1 |
| Yes | 0.84 (0.28–2.49) | 1.09 (0.25–4.87) |

*The model incorporated all variables listed in the table.

BMI, body mass index; CAP, community-acquired pneumonia; CI, confidence interval; COPD, chronic obstructive pulmonary disease; OR, odds ratio.

Supplementary Figure 1. Flowchart showing participant selection in the sensitivity analysis using self-administered respiratory disease status
